# Quality of Life of Children with Short Bowel Syndrome from Patients’ and Parents’ Points of View

**DOI:** 10.3390/children11050536

**Published:** 2024-04-30

**Authors:** Charlotte Kießling, Lucas M. Wessel, Judith Felcht, Cornelia I. Hagl, Michael Boettcher, Rasul Khasanov

**Affiliations:** 1 Department of Pediatric Surgery, University Medical Center Mannheim, Heidelberg University, Theodor-Kutzer-Ufer 1-3, 68167 Mannheim, Germany; 2 Department of Child and Adolescent Psychiatry and Psychotherapy, St. Joseph’s Hospital Berlin Tempelhof, Wüsthoffstraße 15, 12101 Berlin, Germany; 3 Carl Remigius Medical School, Infanteriestraße 11a, 80797 München, Germany

**Keywords:** short bowel syndrome, bowel resection, quality of life, KINDL, children

## Abstract

Despite limited research, existing studies using generic quality of life (QOL) tools indicate decreased physical health and compromised emotional functioning in children with IF. This study investigates QOL in children with short bowel syndrome (SBS) and its determinants. The study included 57 pediatric patients with SBS treated at Mannheim’s University Hospital between 1998 and 2014. To evaluate QOL, the KINDL questionnaire was used. Three age-specific questionnaire variants were employed, and parental proxy reports were collected. Most patients underwent intestinal lengthening procedures, with varying primary diagnoses. A comparison with healthy children from the patient’s perspective revealed no difference but from the parent’s perspective showed lower QOL in SBS patients, especially regarding physical and mental well-being. QOL varied with age, with 7–10-year-olds reporting the lowest scores. Several factors, including independence from parenteral nutrition and the presence of a complete colon, positively influenced QOL. The independence of parenteral nutrition and the presence of a complete colon positively influenced QOL. The Bianchi technique for intestinal lengthening has also shown promise but needs further research. The observation sample in this study is too small to generalize about the whole population of SBS patients. However, this study shows that many health and treatment factors affect QOL, and a large multicenter study is necessary. Our findings underline the importance of appropriate psychological support for children with SBS and their families.

## 1. Introduction

Over the past few decades, outcomes in children with intestinal failure have improved significantly. As result, the focus has turned to other relevant outcomes, including quality of life. Nowadays, relatively few studies with limited numbers of patients have addressed this issue. According to published data, children with intestinal failure (IF) have poorer physical health as measured by health-related quality of life (HRQOL) tools. Dependence on parenteral nutrition (PN) in patients with short bowel syndrome is also associated with impaired emotional functioning. Social dysfunction is common from the parents’ view and in older children. HRQOL quantifies the many factors influencing their functioning and well-being and is a multidimensional concept consisting of physical and psychosocial (including emotional, cognitive, and social) health dimensions. It is based on patient self-reports, although reports by parents are a useful adjunct, particularly for very young or critically ill children [1].

The HRQOL in adults with IF and short bowel syndrome (SBS) is already well studied, and special tools like “disease-specific Short Bowel Syndrome-Quality of Life (SBS-QoL) scale” have been developed [2]. Abdominal pain, loose stools, poor eating habits, and fatigue affect the quality of life for adults. Frequent hospital visits can also contribute to this. Additionally, financial difficulties, anxiety, and depression can impact other family members. The administration of PN at home can be complex and time-consuming. Central lines and stomas also affect body image, make dressing difficult, and disrupt leisure activities [1,3,4]. Apart from the factors already mentioned, kidney stones and impaired kidney function, anemia, infections, fractures caused by reduced bone mineral density, and other such conditions may also affect HRQOL [1]. The QOL in children with IF and SBS has been investigated less systematically. To date, there have been relatively few studies that have addressed these issues, with limited numbers of patients and varying methodologies [1]. The impact of SBS and IF on children and their families is huge, and HRQOL needs to be studied precisely. 

To better assess the impact of SBS and its therapeutic options on quality of life of children and adolescents, we studied HRQOL in children with SBS from patients’ and parents’ points of view.

## 2. Materials and Methods

The study included patients who were treated in the department of pediatric surgery at the University Hospital of Mannheim, with a diagnosis of short bowel syndrome between 1998 and 2014, and who were not older than 17 years.

The study was approved by the local ethics committee (2014-586N-MA).

It is an exploratory study. In terms of the parameters collected, particular emphasis was on the treatment of short bowel syndrome. It was determined whether and which type of intestinal lengthening procedure was performed and whether parenteral nutrition was currently necessary. Moreover, the influence of lengthening procedures on the health-specific quality of life was determined. In addition, data about the individual medical history of the children and adolescents were collected in order to identify other important influencing factors. KINDL questionnaires were sent to the children and their patients to assess health-related quality of life. The child completed the age-appropriate questionnaire, and the parents rated their children’s quality of life. In addition to the KINDL questionnaires, a questionnaire on current developmental and nutritional status was sent.

### 2.1. Assessment of Quality of Life with the KINDL Questionnaire

The KINDL^R^ questionnaire, developed in 1994 by Prof. Monika Bullinger [5], is a validated instrument [6] to assess the quality of life of children and young people.

Three Variants of the KINDL questionnaire were used in three age groups:

- “Kiddy” questionnaire for 4–6-year-old children;
- “Kid” questionnaire for 7–13-year-olds;
- “Kiddo” questionnaire for adolescents aged 14–17 years.

The parent questionnaire was used in two versions: one for 4- to 6-year-old children and one for 7- to 17-year-old children.

The questionnaires for children aged 4–6 years include 12 items; the other versions of the KINDL questionnaire contain 24 items that are divided into six different dimensions: (1) physical well-being, (2) emotional well-being, (3) self-esteem, (4) family, (5) friends, and (6) everyday functioning (school or nursery school/kindergarten).

Another component of the KINDL questionnaire is the “disease” module. Six items are presented here that are intended to precisely assess the quality of life concerning the existing illness and thus reflect its influence on the patient’s life. The questionnaires were analyzed using SPSS 23, as described earlier [7]. The data from KINDL questionnaires were transformed to a scale from 0 to 100, which can reflect the children’s quality of life in an easily comparable way. The value 100 indicates the best possible result. The lower the value, the lower the quality of life assessed.

Among the most commonly used instruments for children are the Child Health and Illness Profile (CHIP) [8], the KIDSCREEN-52 [9], the KINDL [5], the Pediatric Quality of Life Inventory (PedsQL) [10,11,12], and the DISABKIDS [13,14]. For our study, we chose the KINDL^R^ questionnaire, which is a measure of HRQoL in healthy and ill children and adolescents (aged 3 years and older) and has been rated as a good and reliable questionnaire. As our study was conducted in Germany on German-speaking families, the advantage of the KINDL for us was that the questionnaire was developed in Germany and validated for the German language. However, one of the most important factors was that unlike PedsQL and CHIP, there are standardized scores for children in Germany, which we used to compare with our results. It measures the HRQoL of children and adolescents in a way that is appropriate for children and their developmental stage at that age. The KINDL-R contains scales for physical, emotional, family, social, and school-related well-being and self-esteem. Several studies have demonstrated its good psychometric characteristics, such as the high reliability of most of its dimensions (Cronbach’s alpha > 0.70) and its good performance in discriminating between different clinical diagnoses. In addition, standardized values for Germany are available for comparison [15]. The results of the QoL survey were compared with those of healthy children from the Child and Adolescent Health Survey to see if and how SBS affects patients’ quality of life [6].

### 2.2. Healthy Population for Comparison

Between 2003 and 2006, the Robert Koch Institute carried out the Child and Adolescent Health Survey, KiGGS for short. The quality of life of 14,836 children and young people nationwide was determined using the KINDL questionnaire. The study aimed to collect reference data and thus standard values for children and young people living in Germany. These were collected for children’s quality of life from the perspective of children aged 11 to 17 years and for children’s quality of life from the parents’ perspective for children aged 3–17 years. The reliability (Cronbach’s alpha = 0.85) and validity could be confirmed at 79.

The average total quality of life for children aged 11 to 17 was 72.6 points, with boys having a slightly higher quality of life than girls (71.2 to 73.9). From the parent’s perspective, the total quality of life of 3–17-year-olds was 76.9 points, but the gender differences were smaller: 76.9 points for girls and 76.8 points for boys.

Furthermore, the study provided data for the subscales of the KINDL questionnaire, all divided into gender and age groups. We used these as reference values for the present study to compare healthy children with pediatric patients suffering from SBS.

### 2.3. Statistical Methods

The statistical analysis was carried out using the statistics program SAS version 9.4. Unpaired *t*-test was carried out on normally distributed data. For non-normally distributed data, the Mann–Whitney U test was used. Statistical significance was accepted when the *p*-value was 0.05 or less (* *p* < 0.05).

## 3. Results

An information letter and questionnaires were sent to the families by post. After completing the questionnaire, parents were asked to return it by post. Twenty-three out of fifty-seven families (40% response rate) completed the questionnaire. There were fourteen boys and nine girls. Fourteen of the children and adolescents (60.9%) underwent intestinal lengthening procedures. LILT (longitudinal intestinal lengthening and tailoring) according to Bianchi was used in nine patients, the STEP (serial transverse enteroplasty procedure) technique in three, and both in two.

As a primary diagnosis at birth, nine children had gastroschisis, seven suffered from NEC (necrotizing enterocolitis), three had intestinal atresia, and two patients had volvulus. The two remaining patients had rarer causes (diaphragmatic hernia and cloacal exstrophy). Exact data on the primary operation were available for 19 children and adolescents. Data on the ileocecal valve were available in 17 cases: in 5 cases, the ileocecal valve was preserved, and in 12 cases, it was resected.

At the time of the survey, 13 of the 23 patients (57%) were dependent on parenteral nutrition. The weight determination showed an average value around the 15th percentile. Twelve children were above the 10th percentile, and nine children were below the 10th percentile.

A nursery school attended by three children, an elementary school by nine children, a school with special needs education by five children, and a high school by six children.

### 3.1. Health-Related Quality of Life of the Patients with SBS

#### 3.1.1. Comparison of the Quality of Life with Norm Values

In order to identify whether and how short bowel syndrome influences patients’ quality of life, the results of the quality of life survey were compared with those of healthy children from the 2007 Child and Adolescent Health Survey [6].

**Figure 1:**
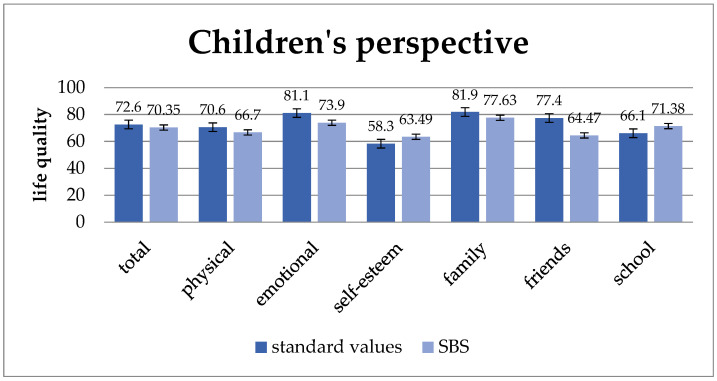
Quality of life from the children’s perspective compared to standard values. The quality of life for short bowel patients exhibits lower scores than standard values across the total quality of life and subunits of physical, mental, family, and friend-related well-being, despite not being statistically significant. Values: mean and standard deviation.

The health-specific quality of life of short bowel patients is lower than that of healthy children and adolescents in terms of “total quality of life” and the subunits “physical well-being”, “mental well-being”, “family well-being”, and “well-being in relation to friends”. However, the quality of life of the subunits “self-esteem” and “school well-being” is rated higher than that of the reference group (Figure 1). However, statistically significant differences could be proven neither in the total quality of life nor in the subunits when comparing the data from short bowel patients with those from healthy children.

**Figure 2:**
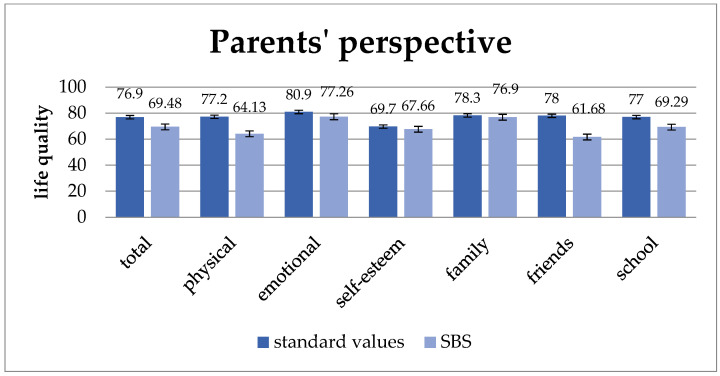
Quality of life from the parents’ perspective compared to standard values. From the parents’ perspective, their children’s quality of life is lower than that of healthy peers, with statistically significant differences in “total quality of life” (*p* = 0.009), “physical well-being” (*p* = 0.007), “well-being in relation to friends” (*p* = 0.001), and “school well-being” (*p* = 0.008). Values: mean and standard deviation.

Parents whose children suffer from short bowel syndrome rate their children’s quality of life in average lower than that of healthy children. As shown in Figure 2, this can be seen in total quality of life and all subunits. The total quality of life (*p* = 0.009) as well as the sub-units “physical well-being” (*p* = 0.007), “well-being in relation to friends” (*p* = 0.001), and “school well-being” (*p* = 0.008) were statistically significantly lower than these of healthy children.

#### 3.1.2. Quality of Life Depending on Age

**Figure 3:**
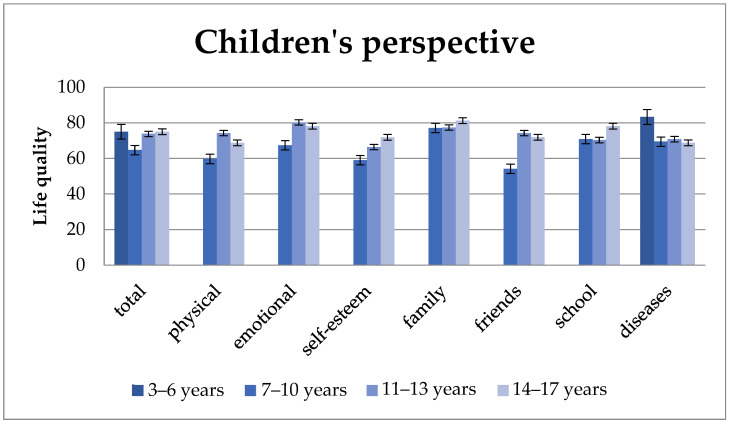
Quality of life from the children’s perspective by age group. Quality of life varies across age groups, with younger children (7–10-year-olds) typically reporting lower scores across most aspects except for “school well-being” and “disease-related well-being”, while “total quality of life”, “self-esteem”, and “family well-being” tend to improve with increasing age. Values: mean and standard deviation.

Health-related quality of life varies according to age. For “total quality of life” and almost all subcategories (exceptions are “school well-being” and “disease-related well-being”) from the perspective of children, 7–10-year-olds report the worst results. This is also reflected in the information provided by parents. With increasing age, the “total quality of life” as well as the subunits “self-esteem” and “family well-being” also increase (Figure 3). School “well-being” is an exception in that 7–10-year-olds achieve slightly better results compared to 11–13-year-olds.

**Figure 4:**
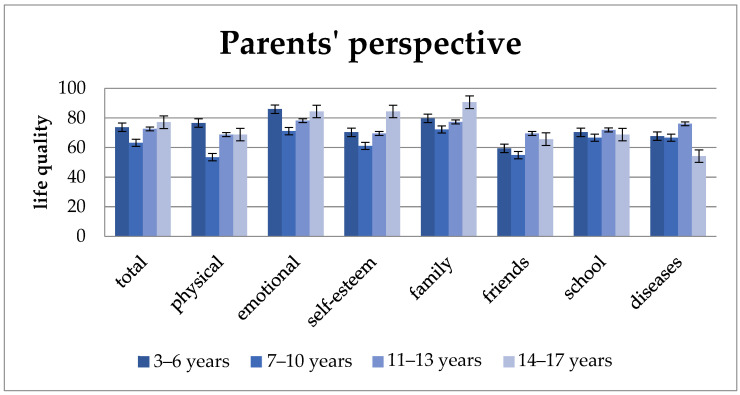
Quality of life from the parents’ perspective by age group. Quality of life ratings are lower for 7–10-year-olds compared to other age groups, with 3–6-year-olds generally rated higher; total quality of life tends to increase from age seven onwards, except for specific dimensions like “well-being in relation to friends” and “school well-being”. Values: mean and standard deviation.

As Figure 4 shows, parents’ reports follow similar trends. Here, parents consistently report the lowest quality of life values for 7–10-year-old children compared to other age groups. The parents of 3–6-year-old children rate their children’s quality of life higher than that of 7–13-year-old children. They only yield a lower result in the subunits “well-being in relation to friends” and “school well-being”. For the total quality of life as well as for most subunits (“physical well-being”, “psychological well-being”, “self-esteem”, and “family well-being”), the quality of life increases from the age of 7; only for the dimensions “well-being in relation to friends” and “school well-being” do the values of 14–17-year = old children fall back below those of 11–13-year-old children.

The additional module, “disease”, is clearly rated best from the perspective of children aged 3–6 years. Afterwards, the results are similar, with children aged 14–17 years reporting the lowest levels. Regarding parental information, in contrast to their children, the highest results occur between the ages of 11 and 13 years, but the lowest results also occur among 14–17-year-old children.

#### 3.1.3. Differences in Age Grouping with Healthy Children

**Figure 5:**
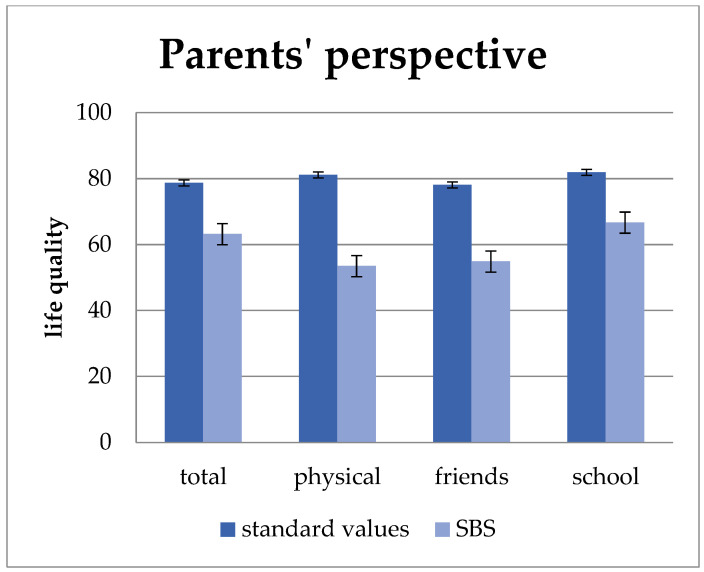
Quality of life from the parents’ perspective compared to standard values divided by the age group 7–10 years. SBS patients are rated significantly lower in “total quality of life” (*p* = 0.003), “physical well-being” (*p* = 0.003), “well-being in relation to friends” (*p* = 0.006), and “school well-being” (*p* = 0.02) than their healthy peers. Values: mean and standard deviation.

When dividing the children and adolescents into four age groups (3–6 years, 7–10 years, 11–13 years, and 14–17 years) and re-testing for significant differences in the reference values of healthy children, it is noticeable that the discrepancy with healthy children is particularly striking at the age of 7–10 years (Figure 5). When comparing the results in this age group, one observes the significantly worse quality of life assessments in short bowel patients from the parents’ point of view in the areas of “total quality of life” (*p* = 0.003), “physical well-being”, (*p* = 0.003) “well-being in relation to friends” (0.006), and “school well-being” (*p* = 0.02). No statistically significant differences could be proven in the other age groups. 

### 3.2. Influence of the Collected Parameters on the Total Quality of Life

#### 3.2.1. Influence of the Type of Intestinal Lengthening Procedures on the Quality of Life

**Figure 6:**
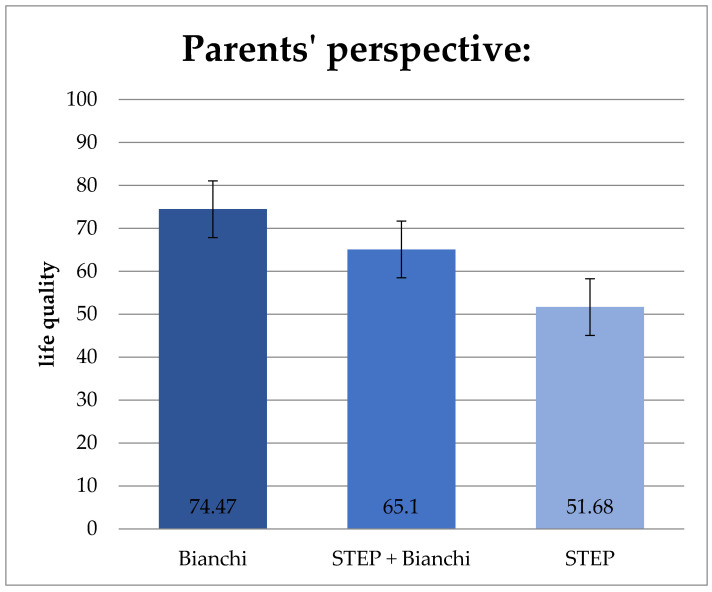
Influence of intestinal lengthening surgery on the total quality of life from the parents’ perspective. The average quality of life of patients who underwent the Bianchi procedure was significantly higher than the STEP procedure (*p* = 0.039). Values: mean and standard deviation.

The total quality of life is significantly different from the parents’ perspective in patients who underwent lengthening procedures according to Bianchi and STEP (*p* = 0.039). As shown in Figure 6, the average quality of life of children who received Bianchi surgery is 74.47 points; for children who have had STEP surgery, the average is 51.68 points, and for patients with both techniques, it is 65.1 points (Figure 6).

#### 3.2.2. Influence of the Ileocecal Valve on Quality of Life

**Figure 7:**
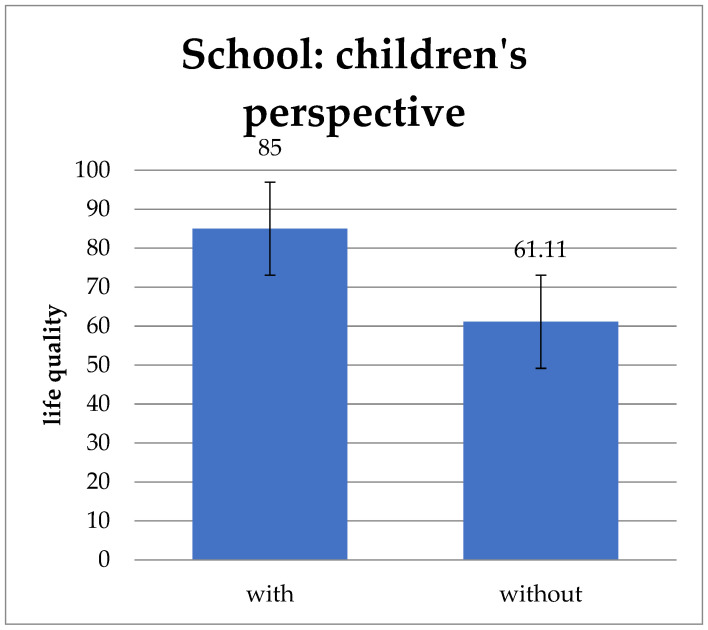
“School well-being” from the children’s perspective divided into with and without ileocecal valve. Children and adolescents with an ileocecal valve have a significantly higher “School well-being” than patients without. Values: mean and standard deviation.

Another parameter is the preserved ileocecal valve (Figure 7). Regarding “school well-being” children and adolescents with an ileocecal valve have a statistically significant better result than patients without (*p* = 0.005).

#### 3.2.3. Influence of the Colon on the Quality of Life

**Figure 8:**
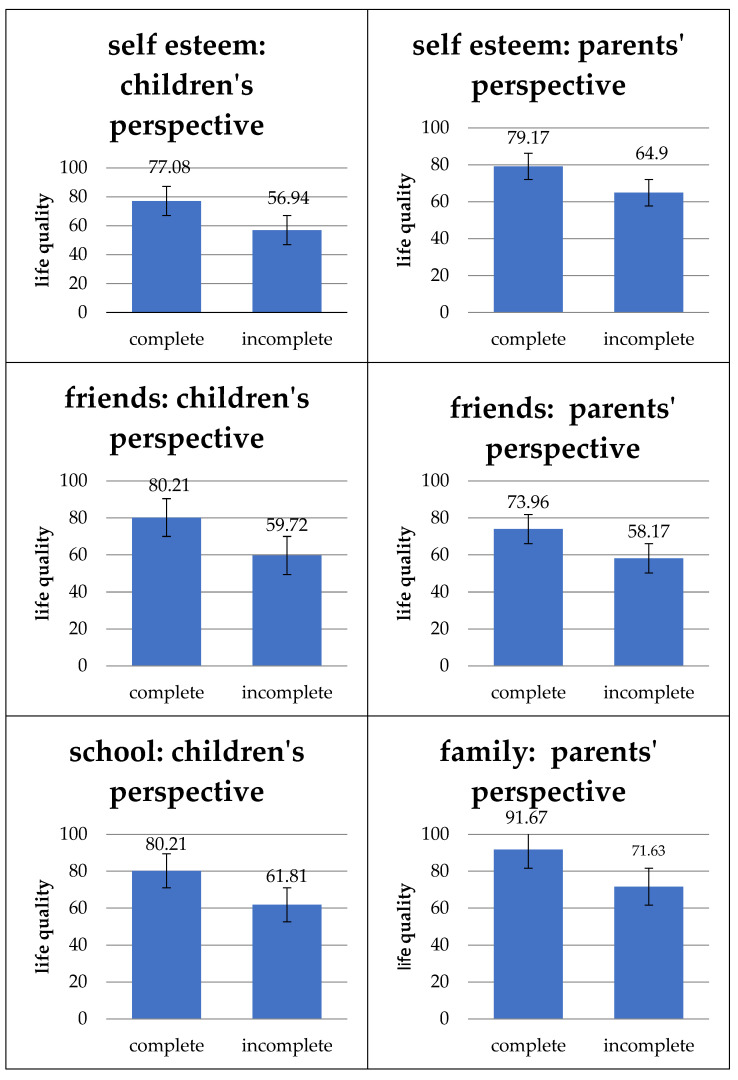
Impact of a complete colon on quality of life and its subunits. Complete colon is significantly associated with higher total quality of life by children (*p* = 0.015) and their parents (*p* = 0.005), as well as the subunits “self-esteem” (children: *p* = 0.008, parents: *p* = 0.008) and “well-being in relation to friends” (children: *p* = 0.032, parents: *p* = 0.042) and from the children’s perspective positively impacting “school well-being” (*p* = 0.007) and from the parents’ viewpoint “family well-being” (*p* = 0.012). Values: mean and standard deviation.

As shown in Figure 8, the study showed that the complete presence of the colon is associated with a statistically significant higher total quality of life from the perspective of the children themselves (*p* = 0.015) and their parents (*p* = 0.005). In addition, a complete colon positively influences the subunits “self-esteem” from the perspective of the children (*p* = 0.008) and their parents (0.008) as well as “well-being in relation to friends” from both perspectives (children: *p* = 0.032, parents: *p* = 0.042). A statistically significant positive effect could also be demonstrated for school well-being from the children’s point of view (*p* = 0.007) and for family well-being from the parents’ point of view (*p* = 0.012). 

#### 3.2.4. Influence of Parenteral Nutrition on Quality of Life

**Figure 9:**
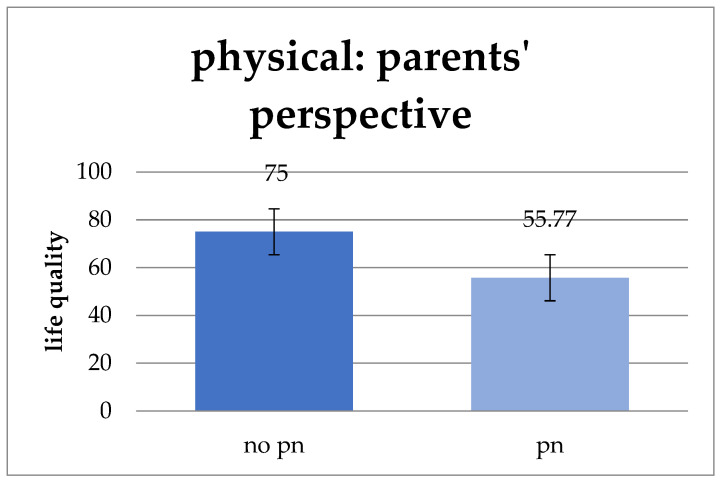
Influence of parenteral nutrition on physical well-being from the parent’s perspective (pn = parenteral nutrition). Parenteral nutrition associated with significantly worse physical well-being (*p* = 0.026). Values: mean and standard deviation.

Parenteral nutrition was associated with significantly worse physical well-being from the parents’ perspective (*p* = 0.026). The average physical well-being of patients with parenteral nutrition is 55.77 points but 75 points for those without this necessity (Figure 9).

## 4. Discussion

The present study shows that SBS is not necessarily associated with a reduced quality of life in affected children. Surprisingly, however, the affected children and their parents assess the quality of life differently. While there are statistically significant differences in the values of the sick children themselves neither in total quality of life nor in the six subunits, the parents of children with short bowel syndrome rate their children’s quality of life as statistically significantly worse than parents of healthy children. Furthermore, having a serious illness does not necessarily mean a lower quality of life. A study of health-related quality of life in children and adolescents with spina bifida showed that there was no difference in health-related quality of life between patients and healthy children and adolescents [16]. Also, in the quality-of-life surveys of patients with esophageal atresia [11,12], asthma and type 1 diabetes [5,17], heart defects [18], cancer [19], or hereditary diseases such as Marfan syndrome [20], it was found that the diseases do not necessarily result in a poorer quality of life.

A study of Finnish children with chronic intestinal failure (most of whom suffered from short bowel syndrome) came to a similar conclusion as the present study. Here, too, the children did not have a worse quality of life than healthy children, leading the authors to state that the children could certainly lead a fulfilling life [21]. That corresponds with results from Sudan et al., according to which pediatric patients who underwent bowel transplants reported feeling that their physical and psychosocial functioning is comparable to normal school children according to their CHO (The Child Health Questionnaire) score [22]. However, using PedsQL4.0 in patients after intestinal transplantation shows significantly lower scores in the subcategory of school functioning and psychosocial health summary score [23]. 

These results raise the question of why diseases that have a lasting impact on the body of those affected are not reflected in their quality-of-life experience. There are several theoretical explanations for this, which are often interrelated. According to Lazarus and Folkman, coping is understood as coping or processing measures when dealing with stress or stressful situations [24]. Serious illnesses often plunge patients into exceptional states in which they automatically develop various coping strategies. Possible examples include denial of the illness and isolation but also increased optimism or religiosity. This can help those affected cope with their illness and positively (but also negatively) impact their quality of life. Another explanation for unexpectedly good results in quality-of-life surveys could be the type of survey itself. Response shift is defined as the change in the meaning of the individual’s quality of life through a change in the internal assessment standards, values, or a redefinition of the individual’s quality of life [25,26]. Thus, it is not the quality of life itself that changes but rather its assessment.

However, there are other studies that are associated with a reduced quality of life by children with SBS. Olieman et al. published a study that describes a lower quality of life of sick children in the total quality of life, the so-called “psychosocial health summary”, as well as in the subunits “physical functionality” and “academic functionality.” In the parent survey, lower values were described in all areas [27]. Also, in the study by Pederiva et al., published in 2018, the patients reported significantly lower values in general quality of life. This also applies to all subunits, with the exception of emotional well-being [28]. However, a different questionnaire (PedsQL) was used in these studies. In intestinal transplant recipients, it has been observed that, like our study, parental assessments of general health and physical functioning differ from the recipients’ own perceptions. Parents tend to perceive a decreased general health and physical functioning level compared to the norms [22,23].

As in the present study, few studies showed that parents had a poorer view of their children’s daily lives: Stahl et al. conducted a study on the health-related quality of life of adolescents with type 1 diabetes in Germany [17], and Musical-Bright et al. published a study on the quality of life of pediatric glioma survivors [19]. 

The birth and care of a seriously ill or disabled child influence the lives of the parents. Sen and Yurtsever describe three phases of parents’ initial reaction after the birth of a disabled child. These are shock, denial, and sadness. The grief is largely based on the parents’ shattered expectations of a “perfect” child. These expectations that they have of their child and of their own life are not met, which leads to anger, depression, fear, and shame and can lead to feelings of guilt [29,30]. 

In addition, the parents’ everyday life is undeniably influenced by the child’s illness. They ensure regular doctor visits and proper home care and, finally, live in constant concern for the well-being of their children, so their siblings, spouse, or themselves are neglected. In many cases, families with seriously ill children are exposed to economic problems, which can lead to further tensions within the family [29,31]. That all results in lower general, mental, and health-related quality of life in parents caring for chronically ill children [32]. Parenteral nutrition at home also results in a reduced quality of life for the parents [33]. Parents of children with short bowel syndrome are exposed to many stressors. They are usually responsible for organizing medical treatment, school, and special therapies (e.g., home parenteral nutrition). Children are usually not aware of this effort. Parents compare their children’s well-being directly with that of other children as well as their own experiences. This seems normal to the children because they do not know a life different from their own, which is experienced by the parents as deficient.

After the age of 10, the total quality of life increases with increasing age from the perspective of children and parents as well as in the subunits “self-esteem” and “family well-being”. This trend can also be seen among parents also in “physical” and “mental well-being”. According to children’s reports, the subunits “physical well-being”, “mental well-being”, and “well-being in relation to friends” initially improve in 11–13-year-olds and then at 14–17 years of age decline again. From the parents’ perspective, this is also the case with “school well-being” and “well-being in relation to friends”.

“Total quality of life” and almost all subcategories (exceptions are “school well-being” and “disease-related well-being”).

Our study shows that 7- to 10-year-old SBS patients rate their quality of life much worse than at other ages. Interestingly, the children after intestinal transplantation show a similar trend: The recipients of intestinal transplants who were between 5 and 10 years old reported significantly lower scores than those between 11 and 18 years old in three domains: global health, general health perception, and family activities [22].

But why do 7–10-year-old children with SBS rate their quality of life so much worse than at other ages? According to classic theories of developmental psychology, the self-image of children in kindergarten and preschool age is still quite incoherent. They usually define themselves through physical characteristics (e.g., “I have brown hair”), activities (e.g., “I like playing football”), or social characteristics (e.g., “I have a big brother”). From primary school age onwards, self-image is increasingly shaped by social comparisons with classmates [34]. One’s own level of functioning is assessed more realistically, and evaluations of others are increasingly incorporated into the self-image [35]. In adolescents, self-image is even more complex compared to younger children. The onset of puberty brings about both physical and intellectual developmental steps [36]. Increased self-reflection leads to a rethinking of norms and values and an increased search for identity [34,36]. It is reasonable to assume that we observe the same mechanism with children with SBS. The studies listed above are consistent with our study in which the 7–10-year age is a particularly vulnerable period in children’s lives and that special care and attention to their psychological well-being is required during this time. 

We analyzed whether surgical therapies such as Bianchi and STEP could impact patients’ quality of life. The tests showed a statistically significant better “total quality of life” after the Bianchi operation from the parents’ perspective (Figure 6). The Bianchi operation also had the highest total quality-of-life scores from the patients’ perspective. However, there was no statistically significant difference. The reasons could lie in a longer experience with the method. Although the Bianchi technique is technically very demanding, it can be assumed that an experienced surgeon can achieve better surgical success [37]. Overall, however, possible sources of error should not be ignored. The number of cases in the study is relatively small, so it would be desirable to study a larger group of patients. It must also be noted that STEP can also be used if the section of the intestine to be lengthened is particularly short or is not suitable for the Bianchi technique, for example, if the duodenum is to be operated on. Therefore, it could be that the patients treated with STEP were already in a more critical situation before the operation, which could explain the low quality of life.

Parenteral nutrition is a cornerstone of the treatment of short bowel syndrome. It enables patients to survive even after extremely extensive intestinal resections [38]. However, parenteral nutrition also involves considerable effort and restrictions. The patients or their caregivers must be instructed in the correct handling and implementation of the infusions and are usually confined to their homes during this time. Preparing, carrying out, and terminating parenteral nutrition takes a lot of time [39] that is not available for other activities. Life-threatening complications cannot be ignored, especially sepsis resulting from infections of indwelling venous catheters, and liver failure can be fatal [38]. For these reasons, we expected that the burden of parenteral nutrition would have a strong negative impact on the quality of life of the children and adolescents in the study group. However, this assumption was not confirmed. Apart from a statistically significantly lower subunit (“physical well-being” from the parents’ perspective), other influences could be proven neither from the perspective of the parents nor from the perspective of the children themselves. In fact, similar results can also be found in the literature. Gottrand et al., in their study of 72 children who were treated with home parenteral nutrition, found no difference between their quality of life and the quality of life of healthy children. However, their parents’ quality of life was definitely negatively affected [33]. Emedo et al. interviewed children who were dependent on parenteral nutrition. They concluded that even though the children were aware of the limitations that the therapy brought with it, they coped very well with it [40]. However, if complications arise during therapy, such as sepsis, because of infections of indwelling venous catheters, the quality of life is again negatively affected [41].

Several studies have found a positive connection between the presence of an ileocecal valve and successful weaning or a shorter duration of parenteral nutrition [42]. The rat model of Barros et al. show that an existing ileocecal valve had a positive influence that could be seen on a histological and molecular level. This could contribute to better intestinal adaptation [43]. In fact, the present study was also able to demonstrate a positive effect of the presence of an ileocecal valve. Children and adolescents whose ileocecal valve was not resected have significantly better school well-being. Since the main function of Bauhin’s valve is to protect the small intestine from bacterial overgrowth, it is not entirely clear why this positive influence is particularly reflected in school well-being. It may contribute to functioning digestion and, through successful weaning from parenteral nutrition, can help children to better cope with school requirements.

An intact colon has been shown to lead to a higher survival rate and makes intestinal adaptation more likely [44]. With an existing colon, the probability of achieving enteral autonomy is higher, and the cases of long-term parenteral nutrition are lower [45,46].

We believe that these effects reflect the results of our study, in which the complete presence of the colon had a positive statistically significant impact on the quality of life from the perspective of the children and their parents in the total quality of life and in subunits “self-esteem” and “well-being in relation to friends”. School well-being from the children’s perspective and family well-being from the parents’ perspective were also statistically significantly higher when the colon was present. The complete colon is, therefore, the factor that most clearly influences the total quality of life and its subunits in the present study.

Absence of the colon reduces water, and electrolyte absorption, dehydration, electrolyte deficiency, shortened intestinal passage, and diarrhea are the consequences [46]. Patients whose colon is partially or completely missing often require a temporary or permanent anus praeter to maintain intestinal transit. This is stressful for patients, can lead to complications [47], and often triggers feelings of shame in young people. Interestingly, however, it was not the subunit “physical well-being” that was influenced, as would be expected, but rather social activities such as “family well-being”, “well-being in relation to school”, and “friends”. One explanation for this could be that children and parents have essentially become accustomed to the body in a state of illness. However, when interacting with friends and in everyday school life, children with SBS are most likely to be restricted by the consequences of their illness because they may find it more difficult than others to play or participate in normal sports, suffer from diarrhea, or have to use a stoma, even if this does not affect their body image. 

This study has some considerable limitations, which need to be addressed: Small number of observations, considerable heterogeneity in the patient population, a different period of time since disease onset, heterogeneity in the treatment of patients and the therapy, and the possible influence of parents on the patient’s responses create limitations and risks of bias.

It is crucial to note that due to limitations of the study, the results of comparing QOL and the influence of the above-described parameters on QOL could not be transferred to the whole population of SBS patients. However, this study shows that many health and treatment factors affect QOL, and a large multicenter study is necessary. Our findings highlight the importance of appropriate psychological support for children with SBS and their families. 

## 5. Conclusions

Our study showed a favorable assessment of QOL by children with SBS themselves; however, parents rated their children’s quality of life lower than their peers. Children with SBS, especially those aged 7–10 years, presented lower QOL and, together with their parents, needed professional psychological support. Positive factors influencing QOL are independence from PN and a complete colon. The Bianchi technique also showed a positive influence on HRQOL. However, because of the small size of the observation, limitations, and risks of bias, it is not possible to directly transfer the results of the study to the whole population of SBS patients. Our data show that a large multicenter study is required to conduct a confirmatory assessment of the QOL of children with SBS.

## Data Availability

The raw data supporting the conclusions of this article will be made available by the authors on request due to privacy, legal and ethical reasons.
